# Difficult Intraoperative Heparinization Following Andexanet Alfa Administration

**DOI:** 10.5811/cpcem.2019.9.43650

**Published:** 2019-10-14

**Authors:** C. James Watson, Sara L. Zettervall, Matthew M. Hall, Michael Ganetsky

**Affiliations:** * Beth Israel Deaconess Medical Center, Department of Emergency Medicine, Boston, Massachusetts; † Beth Israel Deaconess Medical Center, Division of Vascular and Endovascular Surgery, Boston, Massachusetts

## Abstract

Direct oral anticoagulants are now commonplace, and reversal agents are recently becoming available. Andexanet alfa (AnXa), approved by the United States Food and Drug Administration in 2018, is a novel decoy molecule that reverses factor Xa inhibitors in patients with major hemorrhage. We present a case of a 70-year-old man taking rivaroxaban with hemodynamic instability from a ruptured abdominal aortic aneurysm. He received AnXa prior to endovascular surgery, and intraoperatively he could not be heparinized for graft placement. Consideration should be given to the risks and benefits of AnXa administration in patients who require anticoagulation after hemorrhage has been controlled.

## INTRODUCTION

Apixaban and rivaroxaban are direct oral anticoagulants (DOAC) that inhibit factor Xa (FXa), thereby preventing the conversion of prothrombin to thrombin and inducing coagulopathy.^1^ Based on clinical trials that led to United States Food and Drug Administration (FDA) approval as well as post-marketing experience, DOACs carry a similar risk of life-threatening hemorrhage as compared to vitamin K antagonists such as warfarin.^2^–^5^ Agents for reversal of DOAC-induced coagulopathy have only recently been developed. Andexanet alfa (AnXa) was reported by Lu et al. in 2013 to be a reversal agent for the FXa inhibitors. It is a decoy FXa molecule that avidly binds FXa inhibitors, temporarily inhibiting their anticoagulant effects.^6^ It was also noted in vitro to have a binding affinity for heparin-bound antithrombin, thereby suggesting an effect against heparin-induced coagulopathy.^6^–^8^

AnXa was approved by the FDA in May 2018 under regulations allowing for Accelerated Approval for Biological Products for Serious or Life-Threatening Illnesses (21 CFR 601 Subpart E).^9^ Approval was based on efficacy and safety data from the “Andexanet Alfa, a Novel Antidote to the Anticoagulation Effects of FXa Inhibitors Apixaban and Rivaroxaban” (ANNEXA-A and -R, respectively) trials in healthy volunteers, as well as from preliminary data from the “Andexanet Alfa, a Novel Antidote to the Anticoagulation Effects of Factor Xa Inhibitors” (ANNEXA-4) trial in patients with “acute major bleeding.” ANNEXA-4 showed that AnXa has a marked, albeit transient, impact on the surrogate outcome of median anti-FXa activity among those on apixaban or rivaroxaban who have clinically significant hemorrhage.^10^,^11^ Notably, ANNEXA-4 excluded patients requiring emergent surgery, and identified a 10% 30-day risk of thromboembolic events.^11^ Following FDA approval, guidelines have been published recommending AnXa for the reversal of apixaban and rivaroxaban in patients with major hemorrhage, including those who require emergent surgery.^12^,^13^

A ruptured abdominal aortic aneurysm (AAA) is a rare but rapidly life-threatening illness, accounting for 6274 U.S deaths in 2017.^14^ Survival depends on early identification, effective resuscitation, and rapid access to vascular surgery (VS). Increasingly, ruptured aneurysms are being managed endovascularly.^15^ Somewhat counterintuitively, to reduce thrombotic complications these patients receive intraoperative heparinization once proximal control and hemostasis have been achieved.^16^ For patients taking a DOAC with a ruptured aneurysm, initial emergency department (ED) resuscitation may include reversal of anticoagulation. These decisions should be made in consultation with VS and should consider factors such as hemodynamic stability, concern for ongoing bleeding, and time to obtaining proximal control. It is unclear how treatment with AnXa would impact intraoperative anticoagulation and subsequent thrombotic risk.

## CASE REPORT

A 70-year-old, 85-kilogram (kg) man with a history of atrial fibrillation on rivaroxaban, a known 4.5 centimeter (cm) infrarenal AAA, and remote three-vessel coronary artery bypass graft was transferred to our academic, tertiary referral center from a community hospital with the diagnosis of a ruptured AAA. He had last taken 20 milligrams (mg) of rivaroxaban the morning of presentation. At 2 pm, he developed abdominal pain associated with intermittent ripping sensations into his back. He presented to the outside hospital somnolent, with a systolic blood pressure of 80 millimeters of mercury (mmHg). Computed tomography with angiogram demonstrated rupture of his AAA with an associated retroperitoneal hematoma. He received two units (U) of packed red blood cells (pRBC) prior to transfer.

The patient arrived in our ED at 9:35 pm, appearing ashen and uncomfortable. He had a new oxygen requirement of four liters (L) by nasal cannula, a respiratory rate of 24 breaths per minute, a heart rate of 103 beats per minute, and a blood pressure of 122/85 mmHg. The VS team was emergently consulted and he was taken to the operating room (OR) for endovascular aortic repair (EVAR) at 10:57 pm. In our ED, laboratory studies showed the following: hemoglobin 13.0 grams per deciliter (g/dL) (13.7–17.5g/dL), creatinine 1.1 mg/dL (0.5–1.2mg/dL), international normalized ratio (INR) 2.6 (0.9–1.1), and partial thromboplastin time 28.0 seconds (s) (25.0–36.5 s).

**Figure 1:**
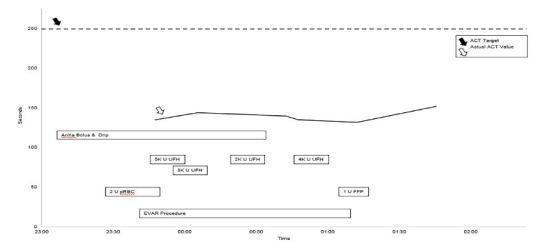
Timeline in operating room. Boxes represent administration of medications and blood products, as well as timing of the surgical procedure. The solid line represents the measured activated clotting time, which never became therapeutic (dashed line). *ACT*, activated clotting time; *AnXa*, Andexanet alfa; *K*, thousand; *U*, units; *UFH*, unfractionated heparin; *pRBC*, packed red blood cells; *FFP*, fresh frozen plasma; *EVAR*, endovascular repair.

Due to his hypotension at the outside hospital, poor skin perfusion, supplemental oxygen requirement, and history of recent rivaroxaban dose with elevated INR qualitatively suggesting coagulopathy and ongoing bleeding, the patient was ordered for low-dose AnXa (400 mg intravenous bolus followed by 4 mg/minute infusion) in consultation with VS. Due to the long preparation time, the AnXa was not immediately available in the ED, and it was started at 11:07 pm by the anesthesiologist in the OR. Two additional units of pRBCs were administered at 11:30 pm and 11:44 pm in the setting of persistent tachycardia to the 120s. A timeline of the operative course is outlined in Figure 1.

Intraoperative heparinization (typically 80–100 U/kg) is used to reduce thromboembolic risk during EVAR.^16^ Intensity of anticoagulation is monitored by activated clotting time (ACT), with target values greater than 250 seconds This patient’s initial ACT by point-of-care assay was 135 at 11:48 pm, approximately 40 minutes after the AnXa bolus and two-hour infusion was started (non-coagulopathic range 80–120 s). After obtaining hemostasis, the VS team proceeded with anticoagulation. The patient was bolused with unfractionated heparin (UFH) 8000 U between 11:49 pm and 12:04 am, and a repeat ACT at 12:06 am was minimally changed (144 seconds). Over the next hour an additional 6000 U UFH were given, and the ACT remained well below the 250-second goal. The hematology service was consulted intraoperatively and recommended transfusing a unit of fresh frozen plasma, which was administered at 1:19 am. For the remainder of the surgery, which ended at 1:46 am, the ACT never exceeded 152 seconds.

The VS team continued with aortic graft deployment despite subtherapeutic heparinization. The aneurysm extended into the right common iliac artery; therefore, to obtain adequate endovascular seal and exclusion of the aneurysm, the endograft’s right limb was extended into the right external iliac artery necessitating coverage of the right hypogastric artery. The left iliac limb terminated just proximal to the left hypogastric artery. Completion angiogram following deployment revealed adequate exclusion of the aneurysm; however, there was evidence of thrombus in the iliac system including the left hypogastric artery.

Following repair, the patient was admitted to the surgical intensive care unit, where he was monitored for four days. He was discharged with no clinical ischemic complications despite extensive thrombosis of his pelvic vasculature. Interestingly, the patient’s INR remained persistently elevated (1.7–2.1) for the remainder of his hospitalization despite remaining off rivaroxaban. The hematology service ordered a mixing study and factor VII (FVII) concentration, which was low at 21% (normal 65–180%), suggesting a congenital FVII deficiency. He was discharged on rivaroxaban 20 mg daily. At hematology clinic follow-up, the patient was well, and his outpatient FVII level remained low (18%) and INR remained high (3.3). This is consistent with congenital FVII deficiency. It is unlikely that the FVII deficiency played a role in the subtherapeutic intraoperative heparinization, as FVII deficiency is a coagulopathy and affects a different point within the coagulation cascade.

## DISCUSSION

We have presented the case of a 70-year-old man on rivaroxaban who suffered a ruptured AAA, received AnXa to reverse FXa inhibitor-induced coagulopathy, and during EVAR was difficult to anticoagulate, thus putting him at increased post-operative thrombotic risk. He sustained thrombosis of his pelvic vasculature without any clinical adverse consequences. This case is highly clinically relevant given the novelty of both the agent and the intraoperative complications at hand; in fact, this case is being published within the clinical pharmacy literature as well with a focus on the pharmacology of the heparin-AnXa interaction and the valuable role of pharmacists in making decisions regarding reversal of anticoagulation.^17^

This case highlights emerging considerations regarding the use of AnXa. This patient underwent an endovascular procedure, the safety profile of which is dependent on intraoperative heparinization. While in vitro data demonstrates inhibition of heparin by AnXa, this relationship has not been clinically studied and is not widely known. AnXa reversibly binds to the heparin-antithrombin complex, rendering heparin ineffective. Therefore, when surgical control of hemorrhage is rapidly available, and intraoperative anticoagulation is important, administration of AnXa may not be warranted. Emergency physicians (EP) should engage their surgical consultants to discuss risks and benefits of using this reversal agent preoperatively.

However, if the EP is caring for a similar patient in a facility without definitive surgical capabilities, AnXa administration may still be a valuable part of the initial resuscitative bundle prior to transfer to a referral center. Currently, AnXa is not widely available in all hospitals, but this is expected to change as manufacturing and distribution improves. AnXa would normalize coagulation parameters during the highest risk period of interfacility transport, while its short half-life would allow it to clear by the time the patient arrives at the referral center. The ANNEXA-A, -R, and -4 studies demonstrated a relative return to expected pre-AnXa anti-FXa activity four hours post-bolus. Ideally, this decision should be made collaboratively between the ED and surgical teams. Notably, if a clinician is faced with needing to anticoagulate a patient after AnXa is administered, a potential strategy would be to use a direct thrombin inhibitor (argatroban or bivalirudin), as the therapeutic effect would be downstream of AnXa in the coagulation cascade. If a patient receives AnXa, it is critical to inform the surgical team, so that they can plan on using one of these alternative agents for intraoperative anticoagulation.

Based on ANNEXA-4, 10% of patients administered AnXa suffered from a thromboembolic event within 30 days of administration.^11^ This is somewhat comparable to the venous thromboembolism rate of 5–7% from other anticoagulant reversal strategies such as prothrombin complex concentrates. Patients requiring surgery with a baseline thrombotic risk creates a difficult decision for the EP regarding the use of any reversal agent.

Ultimately, AnXa is a valuable intervention for the reversal of major hemorrhage in the setting of FXa inhibitor use. Less is understood about the role of AnXa in patients with aortic catastrophe for whom definitive management requires surgery with intraoperative heparinization, and for whom there already exists an intra- and postoperative thrombotic risk. Based on this case, the decision to use AnXa should be made with collaboration between the ED, VS, and pharmacy teams, and should take into consideration the patient’s acute instability, time to surgical control of the aorta, inability to heparinize in the presence of AnXa, and the baseline operative thrombotic risk.

## CONCLUSION

We present a case of a 70-year-old man taking rivaroxaban who suffered a ruptured AAA and received AnXa for stabilization of retroperitoneal hemorrhage, but then could not be adequately heparinized during EVAR. Although it is has been established that AnXa will prevent anticoagulation with heparin, this is not well known within the clinical community. Patients who require heparinization for endovascular surgery may not be appropriate for immediate preoperative AnXa administration.

**CPC-EM Capsule**

What do we already know about this clinical entity?

*Andexanet alfa is a reversal agent for direct Factor Xa inhibitors. It may be valuable in patients with major hemorrhage requiring operative intervention*.

What makes this presentation of disease reportable?

*A patient on rivaroxaban with a ruptured aortic aneurysm received andexanet alfa. This complicated his emergent aortic repair by inhibiting necessary heparinization*.

What is the major learning point?

*Patients requiring endovascular surgery require careful consideration prior to administration of andexanet alfa*.

How might this improve emergency medicine practice?

*Increased communication with vascular surgeons regarding the use of andexanet alfa in endovascular candidates may help prevent operative complications*.
